# Protective Effects of Low-Frequency Magnetic Fields on Cardiomyocytes from Ischemia Reperfusion Injury *via* ROS and NO/ONOO^−^

**DOI:** 10.1155/2013/529173

**Published:** 2013-11-07

**Authors:** Sai Ma, Zhengxun Zhang, Fu Yi, Yabin Wang, Xiaotian Zhang, Xiujuan Li, Yuan Yuan, Feng Cao

**Affiliations:** Department of Cardiology, Xijing Hospital, Fourth Military Medical University, Xi'an, Shannxi 710032, China

## Abstract

*Background*. Cardiac ischemia reperfusion (I/R) injury is associated with overproduction of reactive oxygen species (ROS). Low frequency pulse magnetic fields (LFMFs) have been reported to decrease ROS generation in endothelial cells. Whether LFMFs could assert protective effects on myocardial from I/R injury *via* ROS regulation remains unclear. *Methods*. To simulate *in vivo* cardiac I/R injury, neonatal rat cardiomyocytes were subjected to hypoxia reoxygenation (H/R) with or without exposure to LFMFs. Cell viability, apoptosis index, ROS generation (including O_2_^−^ and ONOO^−^), and NO production were measured in control, H/R, and H/R + LFMF groups, respectively. *Results*. H/R injury resulted in cardiomyocytes apoptosis and decreased cell viability, whereas exposure to LFMFs before or after H/R injury significantly inhibited apoptosis and improved cell viability (*P* < 0.05). LFMFs treatment could suppress ROS (including O_2_^−^ and ONOO^−^) generation induced by H/R injury, combined with decreased NADPH oxidase activity. In addition, LFMFs elevated NO production and enhanced NO/ONOO^−^ balance in cardiomyocytes, and this protective effect was *via* the phosphorylation of endothelial nitric oxide synthase (eNOS). *Conclusion*. LFMFs could protect myocardium against I/R injury *via* regulating ROS generation and NO/ONOO^−^ balance. LFMFs treatment might serve as a promising strategy for cardiac I/R injury.

## 1. Introduction

Acute coronary syndrome, including acute myocardial infarction (AMI), is one of the major causes of morbidity and mortality for patients with coronary heart diseases worldwide [1]. Effective reperfusion therapies like percutaneous coronary intervention (PCI) and coronary artery bypass grafting (GABG) could reduce myocardial ischemia, which in turn minimize myocardial infarction size and improve patients' prognosis [2]. Reperfusion strategies are necessary to resuscitate the ischemic myocardium; however, they may result in paradoxical cardiomyocyte dysfunction and aggravate tissue damage, a process termed as “reperfusion injury” [3]. Thus, an effective strategy to protect heart against ischemia reperfusion (I/R) injury is imperative for successful treatment of coronary heart diseases.

Low-frequency magnetic fields (LFMFs) are considered to be therapeutic and have been started to be applied more and more commonly in medicine. Many studies have demonstrated that LFMFs are capable of affecting a number of physiological and pathological processes. The protective effects of LFMFs on heart tissue against I/R injury have also been widely studied. It is reported that exposure to low-frequency pulsing electromagnetic fields could alleviate cardiac infarction caused by acute permanent ligation of the left anterior descending artery in rats [4]. Kurian et al. also reported that magnetic field preconditioning could enhance cell survival and diminish apoptotic response to simulated I/R injury in H9C2 cells [5]. These results all indicated that LFMFs might play a positive role in cardiac tissue against I/R injury.

In light of these previous studies, our study was designed to investigate whether LFMFs were capable of protecting cardiac tissue from I/R injury and to figure out its underlying mechanism. To simulate *in vivo* cardiac I/R injury, an *in vitro* cardiomyocyte hypoxia-reoxygenation (H/R) model was established and applied.

## 2. Methods

### 2.1. Isolation, Cultivation, and Identification of Cardiomyocytes (CMs)

Primary cultures of cardiomyocytes were prepared from 1–3-day-old neonatal Sprague-Dawley rat hearts (purchased from the Animal Centre of Fourth Military Medical University). Briefly, hearts were excised from neonatal rats (1–3 days old) and rinsed into 1–3 mm^2^ fragments with phosphate buffered saline (PBS). Then, hearts were minced in PBS and digested in 0.1% collagenase I (Sigma, St. Louis, MO, USA) at 37°C for 5 minutes. Supernatant was collected into 15 mL centrifugal tube with 8 mL medium inside. This process was repeated till the tissue was completely dissolved. The suspensions were pooled and centrifuged at 1,000 rpm for 8 min. Cells were resuspended and subjected to differential attachment at 37°C for 1.5 h to remove fibroblasts. CMs were cultured in high glucose DMEM medium (Hyclone, USA) supplemented with 15% (v/v) fetal calf serum (FCS) and 1% penicillin and streptomycin. The culture medium was replenished every 3 days. Primary cultures of cardiomyocytes were positively identified by cTnI (Abcam, USA) staining. Passage 1 cells were used for further studies.

### 2.2. Hypoxia/Reoxygenation Condition

Cardiomyocytes cultured for 6 days were used for the treatment of hypoxia/reoxygenation. For hypoxia, the culture media were replaced by modified hypoxia solution (NaCl 98.5 mmol/L, KCl 10 mmol/L, NaH_2_PO_4_ 0.9 mmol/L, NaHCO_3_ 6.0 mmol/L, CaCl_2_ 1.8 mmol/L, MgSO_4_ 1.2 mmol/L, sodium lactate 40 mmol/L, HEPES 20 mmol/L, and pH 6.8), and a constant stream of water-saturated 5% CO_2_-95% N_2_ was maintained over the cultures for 3 hours. For reoxygenation, the nutrient solution was changed to high glocose DMEM medium supplemented with 15% fetal calf serum under a water-saturated atmosphere of 5% CO_2_-95% air for 3 h.

### 2.3. Application of Low-Frequency Magnetic Fields

Before or after H/R treatment, cardiomyocytes were exposed to low-frequency pulse magnetic fields by MCY-1 Low-Frequency Pulse Magnetic Field Therapeutic Apparatus (Xi'an Century Institute of Measurement and Control Technology, Xi'an, Shaanxi, China). We generated magnetic fields with different amplitudes in a range of 1.5 mT to 6.0 mT and varying sinusoidal currents of 15 Hz or 20 Hz. Eight different parameters of magnetic fields were applied in this experiment: 1.5 mT/15 Hz, 1.5 mT/20 Hz, 3.0 mT/15 Hz, 3.0 mT/20 Hz, 4.5 mT/15 Hz, 4.5 mT/20 Hz, 6.0 mT/15 Hz, and 6.0 mT/20 Hz, respectively. Timing and duration of LFMFs treatment were 1 h, 3 h, and 5 h before or after H/R treatment, respectively. During the experiments, performed in blind manner, the magnetic intensity was monitored by means of a built-in current sensor.

### 2.4. Evaluation of Different LFMFs Conditions

To determine the best conditions of LFMFs, LFMFs of different parameters were applied to CMs before or after H/R treatment as mentioned above. We measured CMs' viability to evaluate the protective effects of different LFMFs conditions. CMs' viability was evaluated by MTT assay, which is based on the transformation of the tetrazolium salt MTT by active mitochondria to an insoluble formazan salt. Cells were plated in 96-well plates at a density of 5 × 10^4^ cells/mL. MTT was added to each well with a final concentration of 0.5 mg/mL, and the plates were incubated for another 4 h at 37°C. Formazan was quantified spectroscopically at 490 nm by spectrophotometer.

### 2.5. Assessment of Apoptosis of CMs

Apoptosis index of CMs was detected by terminal deoxynucleotidyl TUNEL assay using a commercial Cell Death Detection Kit (Roche, Penzberg, Germany) according to the manufacturer's instructions. The index of apoptosis was expressed as the proportion of the TUNEL-positive CMs to the total CMs.

### 2.6. Determination of Oxidative Stress in CMs

NO, ONOO^−^, and O_2_^−^ were measured to indicate the extent of oxidative stress in CMs. The amount of NO, peroxynitrite (ONOO^−^) in CMs was detected by a commercial NO and ONOO^−^ Detection Kit (R&D, USA) according to the manufacturer's instructions. To further demonstrate that the increase of NO production is from eNOS, we used the nonselective inhibitor of eNOS, L-NAME (1 mmol/L, Beyotime Institute of Biotechnology, Nanjing, China), which could suppress NO production *via* deactivating eNOS; then we tested the NO, ONOO^−^ generation and NO/ONOO^−^ balance with or without L-NAME under H/R conditions.

Two different methods were used to measure the amount of O_2_^−^ in CMs. In the first method, a ROS Detection Kit (GenMed Scientifics Inc., USA) was used for quantification according to the instructions. The second method is dihydroethidine (DHE) staining under adaption of previously described methods [6]. Briefly, hydroethidine staining was used to detect the in situ formation of superoxide according to the oxidative fluorescent microtopography. Cardiomyocytes were harvested and incubated with DHE (DHE, 1 : 1000 dilution, Beyotime Institute of Biotechnology, Nanjing, China) at 37°C for 30 minutes. Cardiomyocytes without any treatment were considered as negative control. In positive control group, cardiomyocytes were incubated with 100 *μ*M H_2_O_2_ for 30 min to induce intracellular reactive oxygen species generation. DHE staining was visualized under a confocal microscope (Olympus, Japan). Then, images of cells were analyzed with Image-Pro Plus software version 6.0. The mean fluorescence intensity of each cell and the total cell emission signals per field were calculated for data analysis.

### 2.7. Quantification of NADPH Oxidase Activity

NADPH oxidase activity was measured by lucigenin-enhanced chemiluminescence with a NADPH oxidase kit (GenMed Scientifics Inc., USA) according to the manufacturer's instructions.

### 2.8. Western Blot Analysis

Western blot was performed as follows. CMs from each group were cultured and harvested at appropriate time. Cells were washed by PBS and scraped using lysis buffer. Total proteins were loaded onto an SDS-PAGE gel and transferred electrophoretically to nitrocellulose membranes (Millipore, Billerica, MA, USA). After blocking with 5% skim milk, the membranes then were hybridized at 4°C overnight with the primary antibody, anti-eNOS (1 : 2000; Abcam), anti-eNOS (phospho S1177, 1 : 2000; Abcam), anti-iNOS (1 : 3000; Abcam), and anti-*β*-actin (1 : 2000; Cell Signaling). The membranes were washed with PBS-Tween and further incubated with secondary antibody, horseradish peroxidase-conjugated goat anti-rabbit IgG (Santa Cruz Biotechnology, CA, USA), at 37°C for 60 minutes. The blots were developed using chemiluminescence reagent kit (Millipore) and visualized with UVP Bio-Imaging Systems. Blot densities were analyzed by Vision Works LS Acquisition and Quantity One Analysis Software.

### 2.9. Data Analysis

All data were expressed as mean ± SD and were analyzed using one-way ANOVA followed by Tukey's multiple comparison test for post test. A value of *P* < 0.05 was considered to be statistically significant. All statistical tests were performed using GraphPad Prism software version 5.0 (GraphPad Software, San Diego, CA, USA). 

## 3. Results

### 3.1. Isolation, Cultivation, and Identification of CMs

**Figure 1:**
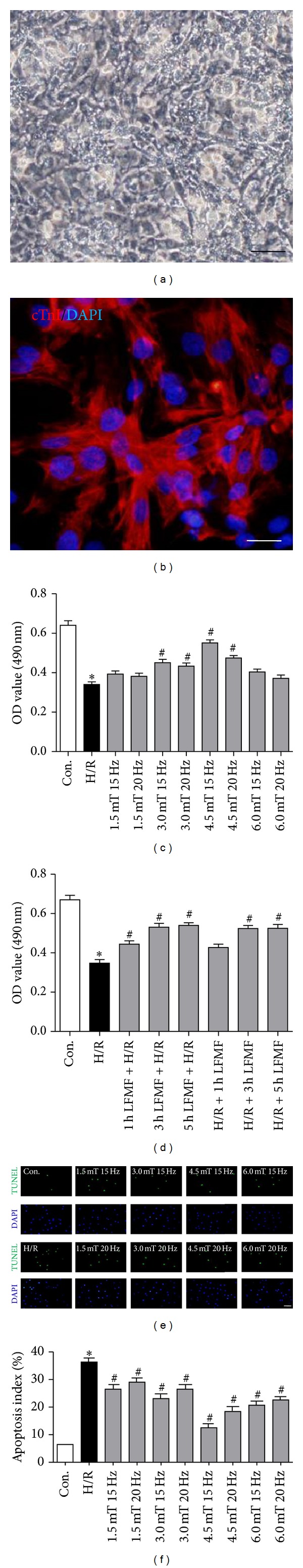
Identification of primary cardiomyocytes (CMs) and optimal low-frequency pulse magnetic fields (LFMFs) conditions. (a) Primary CMs by light microscope (scale bar, 100 *μ*m). (b) cTnI staining of primary CMs by immunofluorescence (red, cTnI; blue, DAPI; scale bar, 50 *μ*m). (c), (d) Cell viability measurement by MTT assays under different LFMFs conditions. *P < 0.05 versus control group; ^#^ *P* < 0.05 versus H/R group. (e) Representative images of immunostaining for apoptotic cells (green, TUNEL; blue, DAPI; scale bar, 100 *μ*m). (f) Quantification of apoptotic cells by Image-Pro Plus software. *P < 0.05 versus control group; ^#^ *P* < 0.05 versus H/R group.

The cells displayed spontaneous contracting with a beating rate of 120–150 beats/min 72 h after isolation. Light microscopy revealed that the spontaneous beating area was mainly composed of relatively large mononuclear cells, approximately 50 *μ*m in diameter, with fusiform or polygon morphology (Figure 1(a)). For further confirmation of CMs, immunofluorescence staining of troponin I (cTnI) was performed.  Figure 1(b) shows positively stained CMs by anti-cTnI. As expected, green fluorescence-labeled cTnI and blue fluorescence-labeled nuclei were observed, indicating the successful isolation and cultivation of CMs.

### 3.2. LFMFs Increased Cardiomyocyte Viability after H/R Injury

It was indicated in the MTT assay that the survival and proliferation rate of CMs remarkably declined after H/R treatment (Figures 1(a) and 1(b)). Exposure to LFMFs of different parameters and durations attenuated this H/R-induced cell suppression and cell death on different levels. Notably, LFMFs exposure to 4.5 mT/15 Hz, 3 h before or after H/R treatment significantly protected CMs against H/R injuries (Figures 1(a) and 1(b)).

### 3.3. LFMFs Decreased H/R-Induced Apoptosis in CMs

To investigate the role of LFMFs in H/R-induced apoptosis in CMs, TUNEL assay was performed. H/R treatment significantly increased the percentage of TUNEL-positive cells. Compared with cells treated with H/R, the LFMFs-treated groups showed a significant decline in apoptotic cells, especially under the condition of 4.5 mT/15 Hz and 3 h before or after H/R treatment (Figures 1(e) and 1(f)). These results demonstrated that LFMFs could exert an antiapoptotic effect on H/R-treated CMs.

### 3.4. LFMFs Exposure Decreased H/R-Induced ROS Production

**Figure 2:**
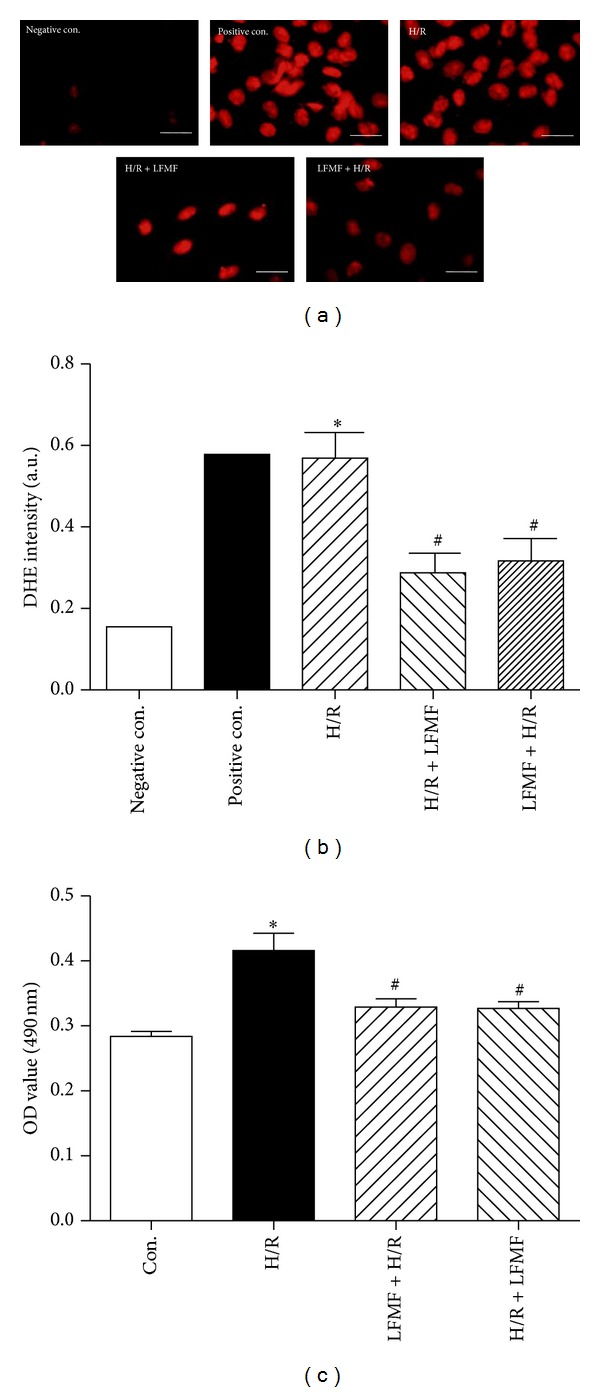
O_2_^−^ as an index of ROS generation in CMs. (a) Representative images of dihydroethidine (DHE) staining of CMs (red, DHE; scale bar, 50 *μ*m). (b) The average fluorescence intensity from five fields was summarized. *P < 0.05 versus control group; ^#^ *P* < 0.05 versus H/R group. (c) O_2_^−^ measurement by commercial ROS Detection Kit. *P < 0.05 versus control group; ^#^ *P* < 0.05 versus H/R group.

ROS is known to play an important role in H/R injuries. In our study, O_2_^−^ was considered as a key marker for ROS. To determine whether LFMFs exposure could attenuate H/R-induced ROS production in CMs, O_2_^−^ production was measured. To make surely true superoxide generation from the cells, both negative and positive control DHE staining images were provided in Figures 2(a) and 2(b). As indicated in Figures 2(a) and 2(b), O_2_^−^ production increased significantly in H/R-treated CMs compared with negative control group (0.401 ± 0.116 *μ*mol/L versus 0.267 ± 0.049 *μ*mol/L, *P* < 0.05). LFMFs + H/R groups, 4.5 mT/15 Hz, 3 h before or after H/R treatment, significantly suppressed O_2_^−^ production (0.326 ± 0.055 *μ*mol/L and 0.324 ± 0.056 *μ*mol/L, resp., *P* < 0.05) compared with H/R group (Figures 2(a) and 2(b)).

### 3.5. LFMFs Exposure Decreased H/R-Induced Cardiac Injury *via* Regulating ROS Production and NO/ONOO^−^ Balancing

**Figure 3:**
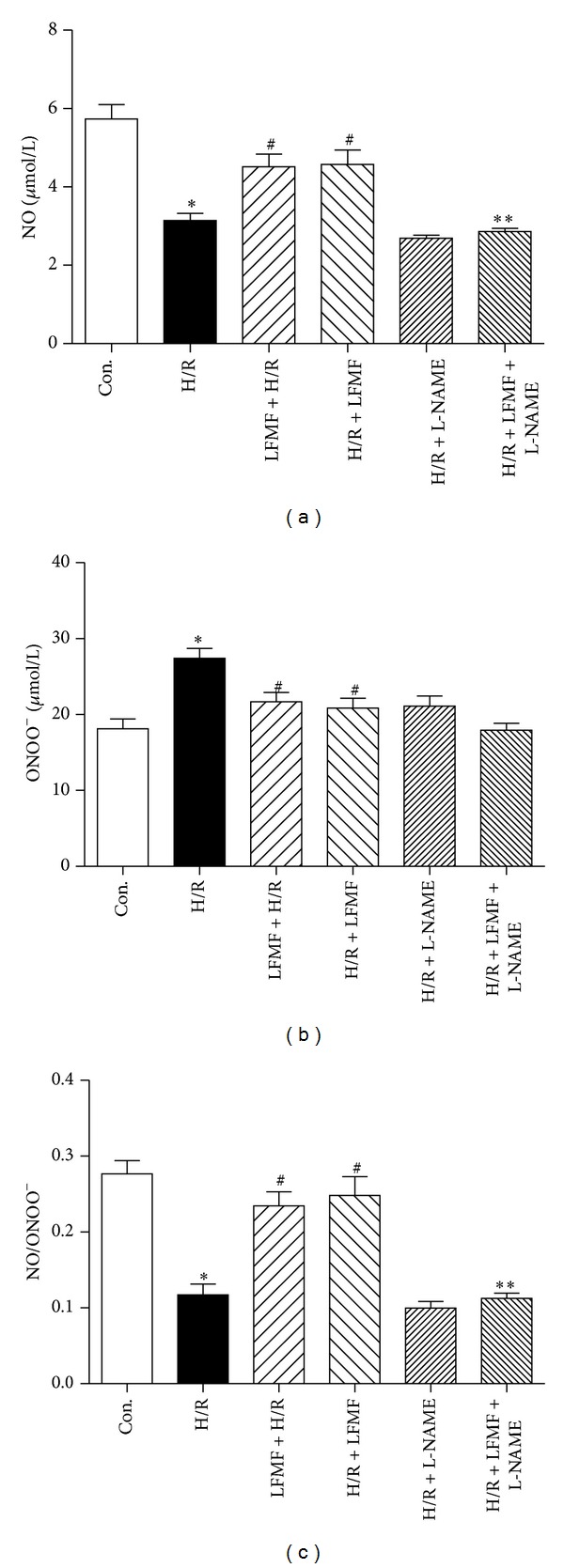
NO, ONOO^−^ production and NO/ONOO^−^ balancing in CMs. (a), (b) NO and ONOO^−^ measurement by commercial NO and ONOO^−^ Detection Kit. *P < 0.05 versus control group; ^#^ *P* < 0.05 versus H/R group; **P < 0.05 versus H/R + LFMF group. (c) Ratio of NO/ONOO^−^ as representative of NO/ONOO^−^ balance. *P < 0.05 control group; ^#^ *P* < 0.05 versus H/R group; **P < 0.05 versus H/R + LFMF group.

NO  production decreased significantly in H/R-treated CMs compared with control group (5.74 ± 2.26 *μ*mol/L versus 3.15 ± 1.06 *μ*mol/L, *P* < 0.05). LFMFs + H/R group and H/R + LFMFs group robustly increased NO production (4.52 ± 1.9 *μ*mol/L and 4.58 ± 1.6 *μ*mol/L) compared with H/R group (*P* < 0.05) (Figure 3(a)). Nevertheless, LFMFs' effect of NO production was eliminated by L-NAME (nonselective inhibitor of eNOS). As demonstrated in Figure 3(a), compared with H/R + LFMF group, NO production in H/R + LFMF + L-NAME was significantly decreased (2.89 ± 1.54 *μ*mol/L versus 4.52 ± 1.95 *μ*mol/L, *P* < 0.05). However, ONOO^−^ production displayed an opposite trend. As indicated in Figure 3(b), ONOO^−^ production increased significantly in H/R-treated CMs compared with control group (27.42 ± 5.74 *μ*mol/L versus 18.12 ± 4.08 *μ*mol/L, *P* < 0.05). LFMF + H/R group and H/R + LFMF group significantly suppressed ONOO^−^ production (21.67 ± 5.45 *μ*mol/L and 20.85 ± 5.77 *μ*mol/L, resp., *P* < 0.05) compared with H/R group. The ratio of NO to ONOO^−^ in H/R group was significantly decreased in comparison with that in control group, while this ratio was increased in LFMF + H/R group and H/R + LFMF group compared with that in H/R group. Similarly, LFMFs' effect on increasing the ratio of NO to ONOO^−^ was reversed by L-NAME (*P* < 0.05).

### 3.6. LFMFs Exposure Decreased H/R-Induced NADPH Oxidase Activity

**Figure 4:**
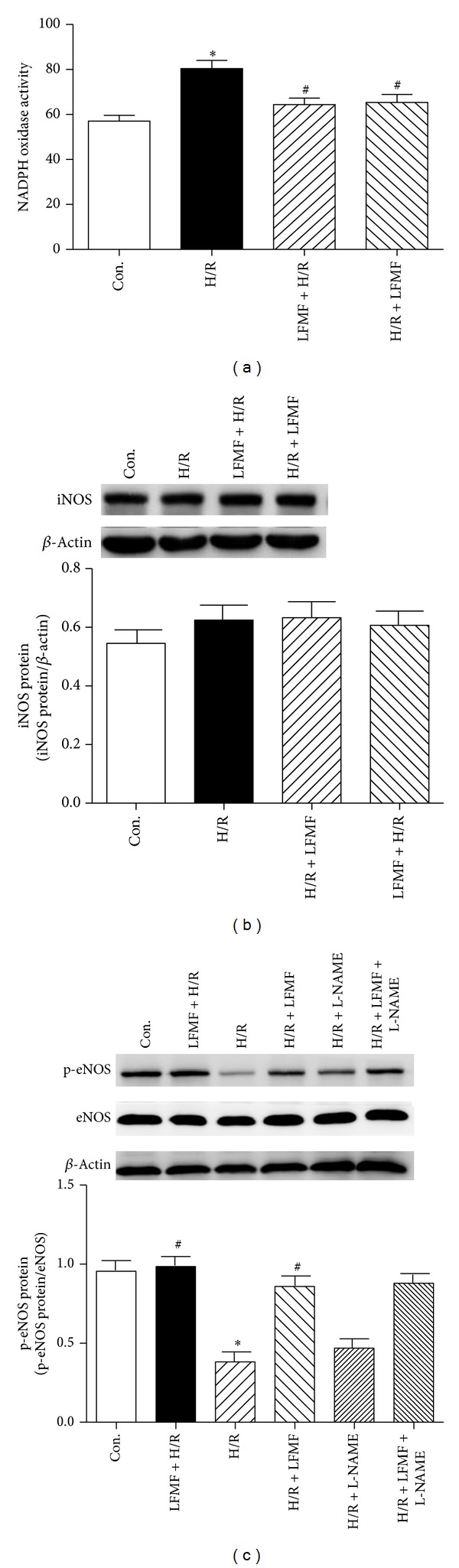
NADPH oxidase activity and phosphorylation of eNOS in CMs. (a) NADPH oxidase activity by commercial NADPH oxidase activity detection kit. *P < 0.05 versus control group; ^#^ *P* < 0.05 versus H/R group. (b) Western blot assay for iNOS expression. No significant difference between groups. (c) Western blot assay for phosphorylated eNOS (phospho S1177) and eNOS expression. *P < 0.05 versus control group; ^#^ *P* < 0.05 versus H/R group.

To further investigate LFMFs' protective role against H/R injuries in antioxidative stress, NADPH oxidase activity was measured. Comparing with control group, NADPH oxidase activity was significantly increased in H/R group (80.33 ± 7.43 RLU versus 54.03 ± 3.21 RLU, *P* < 0.05). However, NADPH oxidase activity was attenuated under LFMFs treatment (64.35 ± 4.68 RLU and 66.29 ± 7.27 RLU, resp., *P* < 0.05) compared with H/R group (Figure 4(a)).

### 3.7. LFMFs Protected CMs against H/R Injuries *via* the Increase of NO Production by the Phosphorylation of eNOS Instead of Inducible Nitric Oxide Synthase (iNOS)

Because eNOS and iNOS are both necessary for NO production and antioxidative reaction, we hypothesized that eNOS played an important role in mediating LFMFs' protection against H/R injuries in CMs. To confirm whether iNOS was associated with LFMFs' protective effect, we tested iNOS protein in each group. Western blot results demonstrated that there was no significant difference among control group, H/R group, and H/R + LFMF group (Figure 4(b)). As indicated in the Western blot results (Figure 4(c)), the phosphorylation of eNOS (phosphor S1177) was declined in H/R group compared with control group, and LFMFs treatment increased the phosphorylation of eNOS (*P* < 0.05). However, L-LAME did not affect the phophorylation of eNOS. Quantification analysis of Western blot results further confirmed this hypothesis (Figure 4(c)).

## 4. Discussion

LFMFs have been reported to suppress apoptosis and enhance cell survival. In the present study, we verified that LFMFs were capable of protecting cardiomyocytes from I/R injury *via* regulating ROS production and NO/ONOO^−^ balance. 

It is recognized that ROS production is elevated by I/R injury and ROS exert crucial effects on I/R injury [7, 8]. ROS, including superoxide radical (O_2_^−^), hydroxyl radical (OH^−^), hydrogen peroxide (H_2_O_2_), and peroxynitrite (ONOO^−^), are able to result in cardiomyocytes' oxidative stress. Oxidative stress could mediate I/R injury by bringing about cardiomyocytes' dysfunction and apoptosis [8]. Lines of evidences have shown that oxidative stress is involved in myocardial damage [9] and antioxidative agents are capable of reducing myocardial injury [10]. Our study revealed similar results. In comparison with control group, the generation of ROS in H/R group was significantly increased which was compromised in the LFMFs-treated groups. This result was in accordance with the variation trend of NADPH oxidase activity. The activity of NADPH oxidase in H/R group was significantly increased compared with control group, and LFMFs exposure suppressed NADPH oxidase activity to a certain degree. This suggested that the amount of O_2_^−^ is positively related to the activity of NADPH oxidase.

LFMFs suppressed the production of ROS, thus exerting a protective role in cardiomyocyte against H/R injury. However, the exact mechanism by which LFMFs decreased the generation of ROS remains unknown. Many researchers supposed that mitochondria played a central role in the increase of ROS generation during I/R [11]. Murphy and Steenbergen considered mitochondrial electron transport as one of the primary sources of ROS in I/R injury [7]. Iorio et al. reported that LFMFs with a square waveform of 5 mT amplitude and frequency of 50 Hz could increase energy generation through regulating mitochondrial oxidative phosphorylation [12]. It is logical to suppose that LFMFs suppress ROS level in cardiomyocytes through regulating mitochondria function. 

In addition to ROS production changes, LFMFs exerted protective effects on cardiomyocytes through upregulating the phosphorylation of eNOS, NO generation and regulating NO/ONOO^−^ balancing. iNOS and eNOS are important for NO production. In the heart, eNOS is constitutively present enzyme in cardiomyocytes, whereas iNOS is absent in the healthy heart, but its expression is induced by proinflammatory mediators [13]. It is necessary to figure out which is responsible for the increase of NO production under LFMFs treatment. Our data indicated that the expression of iNOS protein did not alter under H/R or LFMFs conditions. This manifested that iNOS activation was not the source of the increased NO production under LFMFs treatment after H/R injury. iNOS is currently considered as a primary source for late-phase toxic NO production after myocardial I/R injury. Wildhirt et al. reported that a significant increase in iNOS activity was observed in myocardial regions 48 h after I/R injury [14]. In our experiment, cardiomyocytes protein was harvested immediately after H/R or LFMFs treatment. That may explain why iNOS expression is not elevated in our study. As was demonstrated in our results, the phosphorylation of eNOS and NO production were the minimum in H/R group, and LFMFs exposure restored phosphorylated eNOS and NO generation to some extent. NO could be generated by eNOS and has been reported to exert beneficial effect on cardiac injuries [15, 16], while ONOO^−^, as a component of ROS, has adverse effect on cardiomyocytes viability. The balance of NO/ONOO^−^ plays an important role in cell function and viability. Heeba reported that stains could restore endothelial function by regulating NO/ONOO^−^ balancing *in vitro* [17]. Gao et al. reported that phosphorylation of eNOS and the subsequent increase of NO production contributed significantly to the antiapoptotic effect of insulin on myocardial ischemia-reperfusion Sprague-Dawley rats [18]. Our results were in accordance with the studies mentioned above. As was indicated in our study, LFMFs induced the activation of p-eNOS, resulted in NO generation, and decreased ONOO^−^ production, which shifted the NO/ONOO^−^ balance towards NO. We also found that L-NAME (nonselective inhibitor of eNOS) could abolish the effects of LFMFs on NO production and NO/ONOO^−^ balancing. These testified that eNOS modulated NO/ONOO^−^ balancing, which mediated LFMFs' protective roles in cardiomyocytes. However, iNOS is not involved in LFMFs beneficial effects. Previous studies have reported the favorable effects of LFMFs both *in vivo* and *in vitro*. For example, Yen-Patton et al. demonstrated that pulsed electromagnetic fields could stimulate growth rate of endothelial cells and angiogenesis *in vitro* [19]. However, the effect of LFMFs on organisms and cells remains controversial. Adverse effect of exposure to LFMFs has also been extensively reported. Goraca et al. reported that exposure of rats to ELF-MF (40 Hz, 7 mT, 60 min/day for 2 weeks) resulted in the increase of ROS production in heart tissue and decrease of antioxidant capacity of plasma while exposure to ELF-MF of 40 Hz, 7 mT, 30 min/day for 2 weeks did not alter tissue ROS amount, indicating that the effect of ELF-MF on ROS generation and antioxidative capacity depend on its working time [20]. Emre et al. applied a pulsed square-wave magnetic field at an intensity of 1.5 mT to adult Wistar rats and observed an increase in the levels of oxidative stress indicators and apoptosis in liver samples [21]. Additionally, LFMFs (50 Hz, 1 mT) were also reported to impair cell Ca^+^ homeostasis in spermatozoa [22]. These observed conflicting discrepancies of LFMFs effects may be explained by the different exposure conditions, such as intensity, duration, and frequency of magnetic fields. In our study, LFMFs exposure before or after H/R injury both improved cardiomyocytes viability, but the extent of recovery varied depending on different LFMFs parameters. 

Our study confirmed the protective role of LFMFs in cardiomyocytes against H/R injury, however; the protective effects of LFMFs were not in liner correlation with the intensity and duration of LFMFs exposure. In our present study, limited parameters of LFMFs were applied. There may be a correspondence between particular parameters of LFMFs exposure and their protective effects. To further explore the optimal conditions of LFMFs, especially in clinical practice, further studies are needed.

In conclusion, our present study provides *in vitro* evidence supporting that LFMFs are capable of protecting myocardium from I/R injury *via* regulating ROS production and NO/ONOO^−^ balance. This indicates that LFMFs may serve as a promising therapeutic strategy for cardiac I/R injury.
